# Out of stock: A brief clinical reference for rough equivalency of glucagon‐like peptide‐1 (GLP‐1) ± glucose‐dependent insulinotropic polypeptide (GIP) receptor agonists for A1c and weight reduction in people with type 2 diabetes

**DOI:** 10.1111/1753-0407.13505

**Published:** 2023-11-20

**Authors:** Leigh Perreault, Bryan C. Bergman

**Affiliations:** ^1^ University of Colorado Anschutz Medical Campus Aurora USA

**Keywords:** GLP‐1, GIP/GLP‐1, switching GLP‐1 RA

## Abstract

**Highlights**

- Despite the common practice of switching patients from one medicine to another—to improve efficacy, safety, or tolerability—guidance on how to do so is uncommon. During this time of global shortage of glucagon‐like peptide‐1 receptor agonist (GLP‐1 RA) ± glucose‐dependent insulinotropic polypeptide (GIP) RA therapies, this research letter offers a quick clinical reference of rough equivalency between GLP‐1 ± GIP RA for A1c and body weight reduction in people with type 2 diabetes.

## 1 INTRODUCTION

The recent article in the *Journal of Diabetes* by Jung et al highlights the importance of persistence to glucagon‐like peptide‐1 receptor agonist (GLP‐1 RA) therapy for glucose control and how persistence has improved in recent years.^1^ GLP‐1 RA therapy has gained prominence in guidelines owing to their robust A1c reduction and cardiovascular benefits.^2^ They also harbor patient‐friendly attributes like weight reduction and no increased risk of hypoglycemia. Nevertheless, a new challenge now threatens persistence of GLP‐1 ± glucose‐dependent insulinotropic polypeptide (GIP) RA use: supply.

Twice daily, fixed‐dose injectable exenatide was approved by the US Food and Drug Administration (FDA) as an adjunct to diet and exercise for the treatment of type 2 diabetes in April 2005. Its approval marked the first in the newly created class of GLP‐1 RA. Others soon followed, but their collective utilization remained low (ie, <10% of total prescribed diabetes medicines) until the approval of high‐dose semaglutide (2.4 mg weekly; a GLP‐1 RA) for chronic weight management in June 2021. Higher than expected demand coupled with unforeseen manufacturing hiccups lead to a national shortage in less than 6 months.

Patients and prescribers quickly turned to a lower dose of semaglutide (1 or 2 mg weekly) approved for the treatment of type 2 diabetes, using it off label for chronic weight management. By early 2022, injectable semaglutide—by any brand name—was largely unavailable creating overflow demand for like products, namely dulaglutide (an injectable weekly GLP‐1 RA) and tirzepatide (an injectable weekly GLP‐1 ± GIP RA). It did not take long before these medications also became scarce. By the end of 2022, manufacturers were neither promoting, sampling, or accepting investigator‐initiated studies for these medications. Supply has been forecasted to be back in stock for months now and it is still not clear if or when it will.

The manufacturing shortage of GLP‐1 ± GIP RAs has resulted in rapid switching back and forth between products, largely based on what is available in pharmacies, openly threatening persistence of their use as highlighted by Jung et al. The purpose of this letter to the editor is to provide a brief clinical reference for rough equivalency of approved and commonly used GLP‐1 ± GIP RAs for A1c and weight reduction in people with type 2 diabetes.

## 2 MATERIALS AND METHODS

All data are taken from pivotal trials included in the FDA‐approved prescribing information. Data provided are intended to create the most like comparisons, generally as add‐on to metformin only, avoiding weight increasing medications such as sulfonylureas and thiazolidinediones, as much as possible (trials as add‐on to insulin are not included).^3^, ^4^, ^5^, ^6^, ^7^, ^8^, ^9^, ^10^ Comparisons between products are not the result of head‐to‐head clinical trials. A summary of head‐to‐head trials comparing limited products or doses has been previously published.^11^

## 3 RESULTS

**FIGURE 1:**
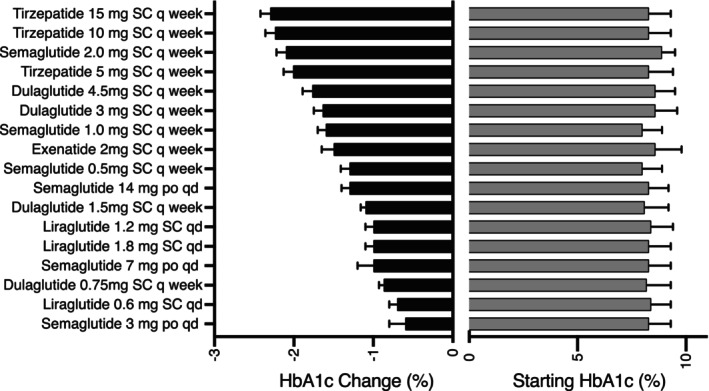
Change in A1c with GLP‐1 ± GIP in people with diabetes. GIP, glucose‐dependent insulinotropic polypeptide; GLP‐1, glucagon‐like peptide‐1; HbA1c, glycated hemoglobin.

**FIGURE 2:**
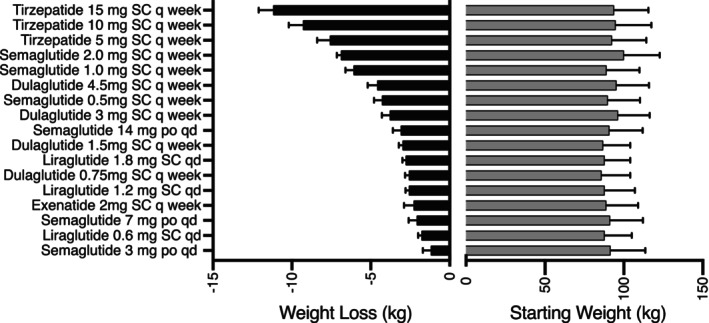
Change in weight with GLP‐1 ± GIP in people with diabetes. GIP, glucose‐dependent insulinotropic polypeptide; GLP‐1, glucagon‐like peptide‐1.

Baseline and change in A1c with GLP‐1 ± GIP RA in people with type 2 diabetes is shown in Figure 1. Baseline and change in weight are shown in Figure 2. Both figures are plotted in order of reported potency to lower A1c or body weight for ease of viewing for clinicians needing to switch patients between products, presuming they are striving for similar A1c and body weight. Trials range from 26 to 56 weeks with A1c and weight mostly stable after 26 weeks. High‐dose dulaglutide (3 and 4.5 mg), moderate‐ to high‐dose semaglutide (1 and 2 mg), and any maintenance dose of tirzepatide (≥5 mg) achieved A1c reduction of ≥1.5%, whereas only semaglutide and tirzepatide consistently achieved a body weight loss of ≥5 kg. Gastrointestinal adverse events (AE) were the most common AE in all trials, ranging from 35% to 65% and scaled with potency for A1c and weight reduction. Warnings and contraindications are largely consistent between products.

## 4 DISCUSSION

GLP‐1 ± GIP RA therapy has rapidly risen up the preferred utilization pathway in guidelines in recent years. First as the preferred injectable before basal insulin owing to noninferiority for A1c reduction without risk of hypoglycemia, next for their cardiovascular benefits, and most recently for their impressive ability to reduce body weight.^2^ Collectively, the glycemic and nonglycemic benefits of these incretin mimetics for people with type 2 diabetes are clear. The unforeseen challenge now is finding them.

The sole purpose of this letter to the editor is to improve persistence of GLP‐1 ± GIP RA use by providing clinicians with a “cheat sheet” of rough equivalency of these medicines for A1c and body weight reduction until the supply issue can be resolved. Many primary care providers, in particular, may not be aware of daily injectable or oral GLP‐1 RA, nor of the many approved maintenance doses that exist. It should be noted, at the time of this letter, no supply issue exists for the daily injectable or oral GLP‐1 RA. Further, it is important to point out that adverse side effects, especially those gastrointestinal in nature, tend to scale with potency of the medications for A1c and weight reduction. Hence, switching to a less potent medication may ease side effects, although individual response is difficult to predict. Conversely, repeating uptitration may not be necessary when switching patients between comparably potent GLP‐1 ± GIP RA who are tolerating their therapy well.

It is not uncommon in the practice of medicine to switch patients from one drug to another to improve efficacy, tolerability and safety. Despite the common practice of switching medications, guidance on how to switch is rare. Currently, there is a global shortage of GLP‐1 ± GIP RA therapies, as well as no guidance on how to switch patients between them. May the current data provide some assistance with the latter.

## FUNDING INFORMATION

No funding supported this work.

## CONFLICT OF INTEREST STATEMENT

Leigh Perreault has received person fees for speaking and/or consulting from Novo Nordisk, Elli Lilly, Boehringer Ingelheim, Neurobo, Medscape, WebMD, and UpToDate. Bryan C. Bergman has no conflicts to declare.

## References

[1] Jung H , Tittel SR , Schloot NC , et al. Clinical characteristics, treatment patterns, and persistence in individuals with type 2 diabetes initiating a glucagon‐like peptide‐1 receptor agonist: a retrospective analysis of the Diabetes Prospective Follow‐Up registry. Diabetes Obes Metab. 2023;25(7):1813‐1822. 36855221 10.1111/dom.15038

[2] Davies MJ , Aroda VR , Collins BS , et al. Management of Hyperglycemia in type 2 diabetes, 2022. A consensus report by the American Diabetes Association (ADA) and the European Association for the Study of Diabetes (EASD). Diabetes Care. 2022;45(11):2753‐2786. 36148880 10.2337/dci22-0034 PMC10008140

[3] Ahren B , Masmiquel L , Kumar H , et al. Efficacy and safety of once‐weekly semaglutide versus once‐daily sitagliptin as an add‐on to metformin, thiazolidinediones, or both, in patients with type 2 diabetes (SUSTAIN 2): a 56‐week, double‐blind, phase 3a, randomised trial. Lancet Diabetes Endocrinol. 2017;5(5):341‐354. 28385659 10.1016/S2213-8587(17)30092-X

[4] Frias JP , Auerbach P , Bajaj HS , et al. Efficacy and safety of once‐weekly semaglutide 2.0 mg versus 1.0 mg in patients with type 2 diabetes (SUSTAIN FORTE): a double‐blind, randomised, phase 3B trial. Lancet Diabetes Endocrinol. 2021;9(9):563‐574. 34293304 10.1016/S2213-8587(21)00174-1

[5] Frias JP , Bonora E , Nevarez Ruiz L , et al. Efficacy and safety of Dulaglutide 3.0 mg and 4.5 mg versus Dulaglutide 1.5 mg in metformin‐treated patients with type 2 diabetes in a randomized controlled trial (AWARD‐11). Diabetes Care. 2021;44(3):765‐773. 33397768 10.2337/dc20-1473 PMC7896253

[6] Frias JP , Davies MJ , Rosenstock J , et al. Tirzepatide versus Semaglutide once weekly in patients with type 2 diabetes. N Engl J Med. 2021;385(6):503‐515. 34170647 10.1056/NEJMoa2107519

[7] Nauck M , Frid A , Hermansen K , et al. Efficacy and safety comparison of liraglutide, glimepiride, and placebo, all in combination with metformin, in type 2 diabetes: the LEAD (liraglutide effect and action in diabetes)‐2 study. Diabetes Care. 2009;32(1):84‐90. 18931095 10.2337/dc08-1355 PMC2606836

[8] Rosenstock J , Perkovic V , Johansen OE , et al. Effect of Linagliptin vs placebo on major cardiovascular events in adults with type 2 diabetes and high cardiovascular and renal risk: the CARMELINA randomized clinical trial. JAMA. 2019;321(1):69‐79. 30418475 10.1001/jama.2018.18269 PMC6583576

[9] Russell‐Jones D , Cuddihy RM , Hanefeld M , et al. Efficacy and safety of exenatide once weekly versus metformin, pioglitazone, and sitagliptin used as monotherapy in drug‐naive patients with type 2 diabetes (DURATION‐4): a 26‐week double‐blind study. Diabetes Care. 2012;35(2):252‐258. 22210563 10.2337/dc11-1107 PMC3263915

[10] Weinstock RS , Guerci B , Umpierrez G , Nauck MA , Skrivanek Z , Milicevic Z . Safety and efficacy of once‐weekly dulaglutide versus sitagliptin after 2 years in metformin‐treated patients with type 2 diabetes (AWARD‐5): a randomized, phase III study. Diabetes Obes Metab. 2015;17(9):849‐858. 25912221 10.1111/dom.12479 PMC5008205

[11] Trujillo JM , Nuffer W , Smith BA . GLP‐1 receptor agonists: an updated review of head‐to‐head clinical studies. Ther Adv Endocrinol Metab. 2021;12:2042018821997320. 33767808 10.1177/2042018821997320 PMC7953228

